# Supplementary material for: Conserved ancestral tropical niche but different continental histories explain the latitudinal diversity gradient in brush-footed butterflies
Source: Nat Commun. 2021 Sep 29;12:5717. doi: 10.1038/s41467-021-25906-8 (PMC8481491; doi:10.1038/s41467-021-25906-8)
Supplement: Supplementary file 3 — Description of Additional Supplementary Files [file 41467_2021_25906_MOESM3_ESM.docx]

Description of Additional Supplementary Files

**File Name**: Supplementary Data 1.

**Description**: List of samples and Genbank accession codes used in this study. Biogeographic distributions are also provided.

**File Name**: Supplementary Movie 1.

**Description**: Animated biogeographic history. Upper plot: map of biogeographic regions used in the DECX model. Dispersal events are depicted by the “arrows” travelling from one area to another. Bottom left plot: Net diversification rate through time for the main biogeographic regions, estimated by the sliding window analysis. Bottom right plot: relative frequency lineages sampled in the tree in each biogeographic region through time.
