# Supplementary material for: Conserved ancestral tropical niche but different continental histories explain the latitudinal diversity gradient in brush-footed butterflies
Source: Nat Commun. 2021 Sep 29;12:5717. doi: 10.1038/s41467-021-25906-8 (PMC8481491; doi:10.1038/s41467-021-25906-8)
Supplement: Supplementary file 6 — Reporting Summary [file 41467_2021_25906_MOESM6_ESM.pdf]

nature research

Corresponding author(s): Nicolas Chazot

Last updated by author(s): Mar 30, 2021

Reporting Summary

Nature Research wishes to improve the reproducibility of the work that we publish. This form provides structure for consistency and transparency in reporting. For further information on Nature Research policies, see our Editorial Policies and the Editorial Policy Checklist.

Statistics

For all statistical analyses, confirm that the following items are present in the figure legend, table legend, main text, or Methods section.

n/a	Confirmed	
☐	☒	The exact sample size (n) for each experimental group/condition, given as a discrete number and unit of measurement
☒	☐	A statement on whether measurements were taken from distinct samples or whether the same sample was measured repeatedly
☐	☒	The statistical test(s) used AND whether they are one- or two-sided<br>*Only common tests should be described solely by name; describe more complex techniques in the Methods section.*
☐	☒	A description of all covariates tested
☐	☒	A description of any assumptions or corrections, such as tests of normality and adjustment for multiple comparisons
☐	☒	A full description of the statistical parameters including central tendency (e.g. means) or other basic estimates (e.g. regression coefficient) AND variation (e.g. standard deviation) or associated estimates of uncertainty (e.g. confidence intervals)
☐	☒	For null hypothesis testing, the test statistic (e.g. F, t, r) with confidence intervals, effect sizes, degrees of freedom and P value noted<br>*Give P values as exact values whenever suitable.*
☐	☒	For Bayesian analysis, information on the choice of priors and Markov chain Monte Carlo settings
☐	☒	For hierarchical and complex designs, identification of the appropriate level for tests and full reporting of outcomes
☒	☐	Estimates of effect sizes (e.g. Cohen's d, Pearson's r), indicating how they were calculated

Our web collection on statistics for biologists contains articles on many of the points above.

Software and code

Policy information about availability of computer code

Data collection	Softwares used for data collection:<br>- VoSeq (Peña and Malm 2012)<br>- Codon Code v.7.1.2 (CodonCode Corporation, www.codoncode.com)<br>- R Core Team (2017). R: A language and environment for statistical computing. R Foundation for Statistical Computing, Vienna, Austria. URL https://www.R-project.org/.
Data analysis	Softwares used for data analyses:<br>- RAxML v.8.2.12 (Stamatakis 2008)<br>- PartitionFinder 2.1.1 (Lanfear et al. 2017)<br>- BEAST 1.8.3. (Drummond et al 2012)<br>- Tracer v.1.6. (Rambaut & Drummond 2018)<br>- LogCombiner 1.8.3 (Drummond et al 2012)<br>- TreeAnnotator 1.8.3 (Drummond et al 2012)<br>- BAMM 2.5 (Rabosky et al. 2013)<br>- DECX (Beeravolu & Condamine 2016).<br>- R Core Team (2017). R: A language and environment for statistical computing. R Foundation for Statistical Computing, Vienna, Austria. URL https://www.R-project.org/.<br>- https://github.com/evogytis/nymphalidae-animation

For manuscripts utilizing custom algorithms or software that are central to the research but not yet described in published literature, software must be made available to editors and reviewers. We strongly encourage code deposition in a community repository (e.g. GitHub). See the Nature Research guidelines for submitting code & software for further information.

Data

Policy information about availability of data

All manuscripts must include a data availability statement. This statement should provide the following information, where applicable:

- Accession codes, unique identifiers, or web links for publicly available datasets
- A list of figures that have associated raw data
- A description of any restrictions on data availability

All sequences used in our manuscript are available on Genbank. All Genbank accession codes can be found in Supplementary Information Table SI. Biogeographic distributions used in our analyses are also available in Supplementary Information Table SI.

Field-specific reporting

Please select the one below that is the best fit for your research. If you are not sure, read the appropriate sections before making your selection.

☐ Life sciences ☐ Behavioural & social sciences ☒ Ecological, evolutionary & environmental sciences

For a reference copy of the document with all sections, see nature.com/documents/nr-reporting-summary-flat.pdf

Ecological, evolutionary & environmental sciences study design

All studies must disclose on these points even when the disclosure is negative.

Study description	The study aimed at: (1) generating a dated super-tree of all Nymphalidae (Papilionoidea) butterflies species sequenced to date, (2) infer dynamics of speciation and extinction rates, (3) estimate historical biogeography of the group and (4) combined previous information to explain the latitudinal and longitunal diversity gradient.
Research sample	Our study includes virtually all Nymphalidae (Papilionoidea) butterflies species sequenced to date (2,866 species). The data combined published sequences available on Genbank and BoLD as well as new data published with this study and now available on Genbank.
Sampling strategy	We included virtually all species of Nymphalidae butterflies sequenced to date. A single representative of each species was included, and chosen based on the number of base pairs available for the different representatives in order to maximise the amount of information available. We based our analyses on 11 gene regions: the mitochondrial COI gene (1473 bp) and the nuclear genes ArgKin (596 bp), CAD (850 bp), DDC (373 bp), EF1a (1240 bp), GAPDH (691 bp), IDH (710 bp), MDH (733 bp), RpS5 (617 bp), RpS2 (411 bp), and wingless (412 bp). These gene regions have been used extensively in molecular systematic studies of Nymphalidae. They were chosen to avoid major gap in data across the tree.
Data collection	Genetic data:<br>We assembled our dataset from two databases: our own VoSeq (Peña and Malm 2012) and BoLD (Ratnasingham & Hebert 2007). From each of these databases we extracted all the individuals classified as "Nymphalidae". In the VoSeq dataset, we filtered multiple hits of species names by keeping only one species representative having the highest number of base pairs available. This first list of taxa was compared to the dataset imported from BoLD, which allowed adding 415 taxa absent from VoSeq for a total of 3,070 taxa. This list of taxa was further cleaned by checking for misspellings of names leading to multiple representatives per species. We also removed all the barcode sequences imported from BoLD that were not associated with a published paper on GenBank. We checked for the validity of all species names, mostly relying on online resources (e.g. http://ftp.funet.fi/pub/sci/bio/life/insecta/lepidoptera/ditrysia/papilionoidea/nymphalidae/). When we encountered a disagreement on taxon assignment we searched for the most recent relevant source of information to decide whether the taxon was included as a valid species or removed. All these sequences were aligned in Codon Code v.7.1.2 (CodonCode Corporation, www.codoncode.com) and uploaded into our VoSeq database. Finally, we used RAxML v.8.2.12 (Stamatakis 2008) on the full dataset, to search for the best scoring topology. This RAxML analysis was used to identify misidentifications, rogue taxa, and other species with unexpected phylogenetic positions likely resulting from human/laboratory error and to check for misalignments. Rogue taxa were removed from the dataset. After these cleaning steps, we ended up with a final taxon sampling of 2,866 species (Table SI. 1).
Timing and spatial scale	Cenozoic and global scale
Data exclusions	We used RAxML v.8.2.12 (Stamatakis 2008) on the full sample of selected species representatives and searched for the best scoring topology. This RAxML analysis was used to identify misidentifications, rogue taxa, and other species with unexpected phylogenetic positions likely resulting from human/laboratory error and to check for misalignments. Rogue taxa were removed from the dataset.
Reproducibility	The list of individuals and associated genbank accession codes used in this study are available in Supplementary Information Table SI. Details about model parameters, partitioning strategies, etc are described in Supplementary Information.
Randomization	There was no experiment requiring group assignments of individuals.
Blinding	Blinding strategy is not relevant for this study. The sampling strategy aimed at maximising the amount of information available at all stages.

Did the study involve field work? ☐ Yes ☒ No

Reporting for specific materials, systems and methods

We require information from authors about some types of materials, experimental systems and methods used in many studies. Here, indicate whether each material, system or method listed is relevant to your study. If you are not sure if a list item applies to your research, read the appropriate section before selecting a response.

Materials & experimental systems

n/a	Involved in the study
☒	☐ Antibodies
☒	☐ Eukaryotic cell lines
☒	☐ Palaeontology and archaeology
☐	☒ Animals and other organisms
☒	☐ Human research participants
☒	☐ Clinical data
☒	☐ Dual use research of concern

Methods

n/a	Involved in the study
☒	☐ ChIP-seq
☒	☐ Flow cytometry
☒	☐ MRI-based neuroimaging

Animals and other organisms

Policy information about studies involving animals; ARRIVE guidelines recommended for reporting animal research

Laboratory animals	This study did not involve laboratory animals.
Wild animals	This study did not involve wild animals.
Field-collected samples	This study was based on specimens not collected for this study specifically but from a variety of collections and databases.
Ethics oversight	There is no ethical issue associated with our study. For example, no collection or exportation of samples were necessary since no field work was performed for this study. It did not involve any living organism (in laboratory or in the wild). All specimens sequenced were done in accordance with the Nagoya Protocol on Access to Genetic Resources and the Fair and Equitable Sharing of Benefits Arising from their Ultilization to the Convention on Biological Diversity.

Note that full information on the approval of the study protocol must also be provided in the manuscript.
